# Matrix metalloproteinase-10 regulates stemness of ovarian cancer stem-like cells by activation of canonical Wnt signaling and can be a target of chemotherapy-resistant ovarian cancer

**DOI:** 10.18632/oncotarget.8645

**Published:** 2016-04-08

**Authors:** Tasuku Mariya, Yoshihiko Hirohashi, Toshihiko Torigoe, Yuta Tabuchi, Takuya Asano, Hiroshi Saijo, Takafumi Kuroda, Kazuyo Yasuda, Masahito Mizuuchi, Tsuyoshi Saito, Noriyuki Sato

**Affiliations:** ^1^ Department of Pathology, Sapporo Medical University School of Medicine, Sapporo, Japan; ^2^ Department of Obstetrics and Gynecology, Sapporo Medical University School of Medicine, Sapporo, Japan; ^3^ Department of Respiratory Medicine and Allergology, Sapporo Medical University School of Medicine, Sapporo, Japan

**Keywords:** ovarian cancer, cancer stem cell, MMP10, chemoresistance

## Abstract

Epithelial ovarian cancer (EOC) is one of the most lethal cancers in females. Cancer stem-like cells (CSCs)/cancer-initiating cells (CICs) have been reported to be origin of primary and recurrent cancers and to be resistant to several treatments. In this study, we identified matrix metalloproteinase-10 (MMP10) is expressed in CSCs/CICs of EOC. An immunohistochemical study revealed that a high expression level of MMP10 is a marker for poor prognosis and platinum resistance in multivariate analysis. MMP10 gene overexpression experiments and MMP10 gene knockdown experiments using siRNAs revealed that MMP10 has a role in the maintenance of CSCs/CICs in EOC and resistance to platinum reagent. Furthermore, MMP10 activate canonical Wnt signaling by inhibiting noncanonical Wnt signaling ligand Wnt5a. Therefore, MMP10 is a novel marker for CSCs/CICs in EOC and that targeting MMP10 is a novel promising approach for chemotherapy-resistant CSCs/CICs in EOC.

## INTRODUCTION

Ovarian cancer is one of the most lethal carcinomas in females. It is often symptomless and it grows and disseminates easily because the ovarian epithelial surface is exposed directly to the abdominal cavity. Ovarian cancer is thus difficult to diagnose in the early stage [1]. It was reported that achievement of optimal debulking, that is, no residual tumor larger than 1 cm in diameter, is an important factor of prognosis [2]; however, achievement of optimal debulking is often difficult due to severe dissemination, adhesion of the tumor and the patient's poor condition. Therefore, adjuvant treatment after the post operation is important for the patient's prognosis. Generally, ovarian cancer is sensitive to platinum agents, and taxan/platinum combined regimens are often used in first-line chemotherapy as adjuvant [3]; however, resistance to platinum reagents is common in late stages. There are several mechanisms underlying resistance to platinum reagents, including changes in cellular uptake, drug efflux, increased detoxification, inhibition of apoptosis and increased DNA repair [4]. Treatment of platinum-resistant tumors is still challenging. A recent clinical trial using bevacizumab, an anti-VEGF antibody, revealed that chemotherapy in combination with administration of bevacizumab improved the progression-free survival rate and objective response rate compared to those with chemotherapy alone but did not improve overall survival [5]. Recent studies have revealed that defect of invasion of cytotoxic T lymphocytes (CTLs) into the tumor lesion and HLA-G expression were predictors of platinum resistance, indicating that lack of immunological surveillance is related to platinum resistance [6, 7]. Expression of PD-L1, an immune checkpoint ligand of HLA-restricted CTL, in ovarian cancer has been reported to be an independent prognostic factor [8], and a clinical study using an anti-PD-1 antibody (nivolumab) for platinum-resistant ovarian cancer has been launched.

Cancer stem-like cells (CSCs)/cancer-initiating cells (CICs) are defined as subpopulation of cancer cells that have high tumor-initiating ability, self-renewal ability and differentiation ability [9]. CSCs/CICs were reported to be resistant to therapies including chemotherapy and radiotherapy, and CSCs/CICs are thus responsible for resistance to treatment and recurrence [10]. Ovarian CSCs/CICs have been isolated by using cell surface markers including CD44 [11], CD24 [12], CD117 [13] and CD133 [14], ALDEFLUOR assay [15] and side population (SP) assay [16]. In this study, we isolated ovarian CSCs/CICs as ALDH^+^ cells by the ALDEFLUOR assay and screened gene expression to analyze the molecular mechanisms of CSCs/CICs and chemoresistance. We identified matrix metalloproteinase-10 (MMP10) as a CSC/CIC-related gene and analyzed its function in the maintenance of CSCs/CICs and chemoresistance.

## RESULTS

### MMP10 is expressed at high levels in epithelial ovarian cancer stem-like cells

**Figure 1:**
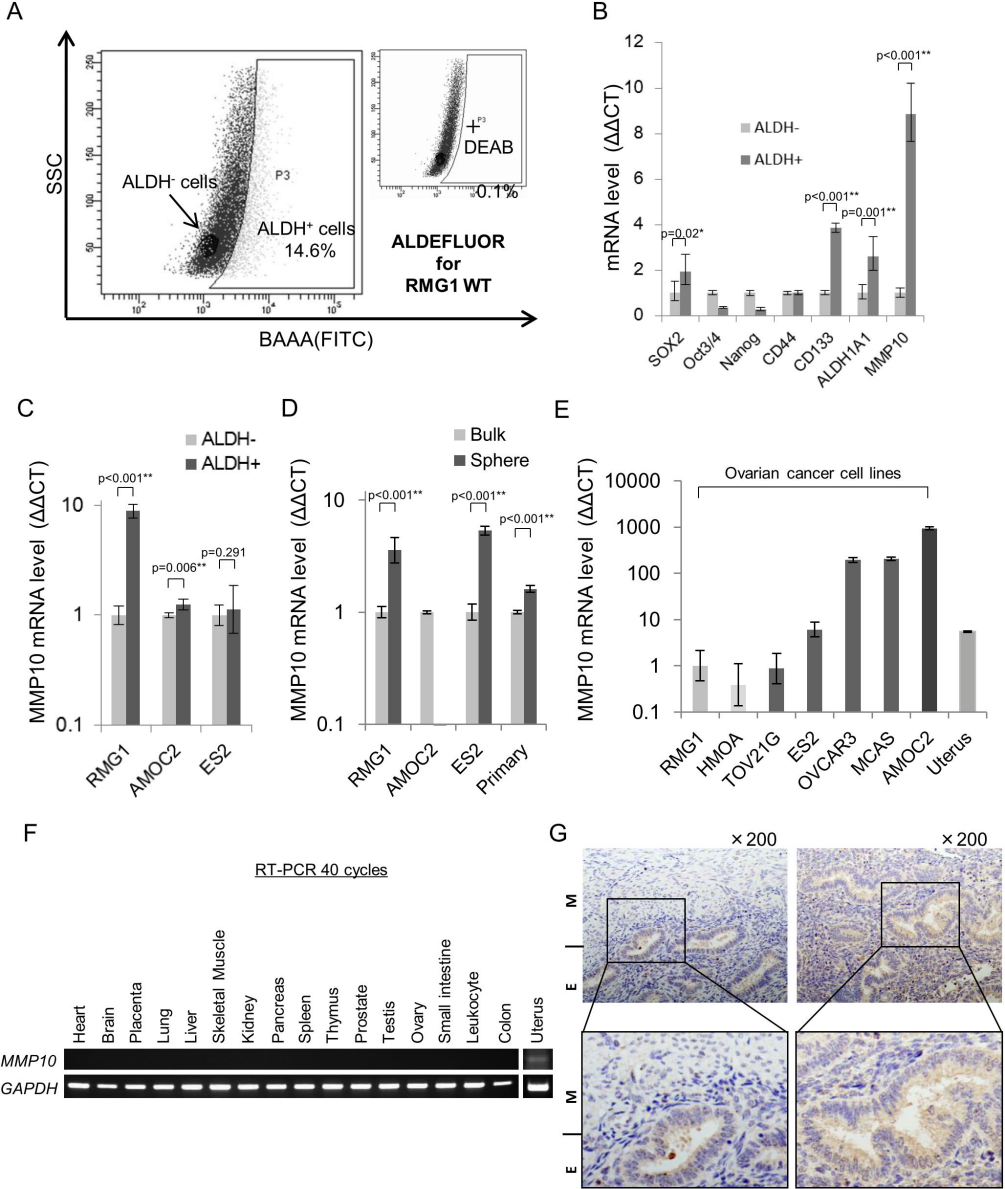
MMP10 expression in ovarian cancer and normal organs (**A**) ALDEFLUOR assay for RMG1 cell line. (**B**) Expression of stem cell-related genes in ALDH^+/−^ cells. Expression levels of stem cell-related genes and MMP10 were determined by quantitative PCR, and differences between ALDH^+^ and ALDH^-^ cells were determined by using the ΔΔCT method. Data are shown as means ± SD. (**C**). MMP10 gene expression level in ALDH^+/−^ cells. Gene expression level of MMP10 was determined by quantitative PCR, and the difference between ALDH^+^ an ALDH^-^ cells of each cell line, RMG1, AMOC2 and ES2, was determined by using the ΔΔCT method. Data are shown as means ± SD. (**D**) MMP10 gene expression levels in bulk and sphere cells. Gene expression levels of MMP10 were determined by quantitative PCR, and the difference between bulk cells (adherent culture cells) and sphere cells (7 days of culture in a sphere-forming conditioned medium) of each cell line, RMG1, AMOC2, ES2 and primary clear cell adenocarcinoma cells, was determined by using the ΔΔCT method. Data are shown as means ± SD. (**E**) MMP10 gene expression levels in cell lines and the uterus. We compared MMP10 gene expression levels in the cell lines and in uterus cDNA. RMG1 was used for a control, and the ΔΔCT method was performed. Data are shown as means ± SD. (**F**) MMP10 gene expression levels in normal organs. (**G**) Immunohistochemical staining of the myometrium and endometrium in the normal uterus for MMP10. The figures on the left are the uterus with an endometrium (E) lesion and a myometrium (M) lesion. The figures on the right are the endometrium of the uterus (upper panel: × 200 magnification, lower panel: enlarged image). All statistical analyses for this figure were performed using bilateral Student's *t* test. *P*-values are shown as follows: *< 0.05, **< 0.01.

We previously reported that highly tumorigenic CSCs/CICs in epithelial ovarian cancer (EOC) can be isolated as ALDH^+^ cells by the ALDEFLUOR assay [15]. In this study, we used the ovarian cancer cell line RMG1 to analyze the transcriptome of CSCs/CICs in EOC. The ratio of ALDH^+^ cells in RMG1 cells was 14.6% (Figure 1A). Total RNAs were isolated from ALDH^+^ cells and ALDH^-^ cells, and analysis was carried out using a cDNA microarray (GSE64539; data uploaded online). Screening was carried out to identify genes that (1) are expressed in ALDH^+^ cells at levels more than 2-fold higher than those in ALDH^-^ cells and (2) are expressed in normal organs at low levels or are not expressed, and one candidate gene, MMP10, was found. To generalize the expression of MMP10 in CSCs/CICs of EOC, we isolated ALDH^+^ cells from different EOC cell lines, AMOC2 and ES2 (Figure S1A). A previous study showed that CSCs/CICs in EOC can be isolated as spheres in a non-adherent culture condition [17]. We thus isolated CSCs/CICs in EOC as spheres from three EOC cell lines (RMG1, AMOC2 and ES2) and a primary EOC sample (Figure S1B). The spheres showed high expression levels of stem cell-related genes, suggesting that CSCs/CICs in EOC were enriched in spheres (Figure S1B). Quantitative RT-PCR analysis (qRT-PCR) revealed that MMP10 was expressed at higher levels in ALDH^+^ cells and spheres than in ALDH^−^ cells and bulk cultured cells with some exceptions (Figure 1C and 1D), indicating that MMP10 is a common EOC CSC/CIC-related gene in EOC.

We then performed qRT-PCR and RT-PCR to examine the expression of MMP10 in EOC cell lines and normal organs. MMP10 is expressed only in cancers and in the uterus among normal organs as estimated by analysis using an online database (biogps.org) (Figure 1E, 1F). Immunohistochemical staining revealed that MMP10 is specifically expressed in endometrium, but not in myometrium (Figure 1G). A microarray datasets of normal endometrium (GSE6364, GSE4888; [18, 19]) showed that MMP10 is expressed mainly in the endometrium of the late secretory phase at a significantly higher level among the menstrual cycle (late secretory phase vs. other phase; *p* < 0.001: Figure S2).

### MMP10 has an essential role in the maintenance of CSCs/CICs in EOC

**Figure 2:**
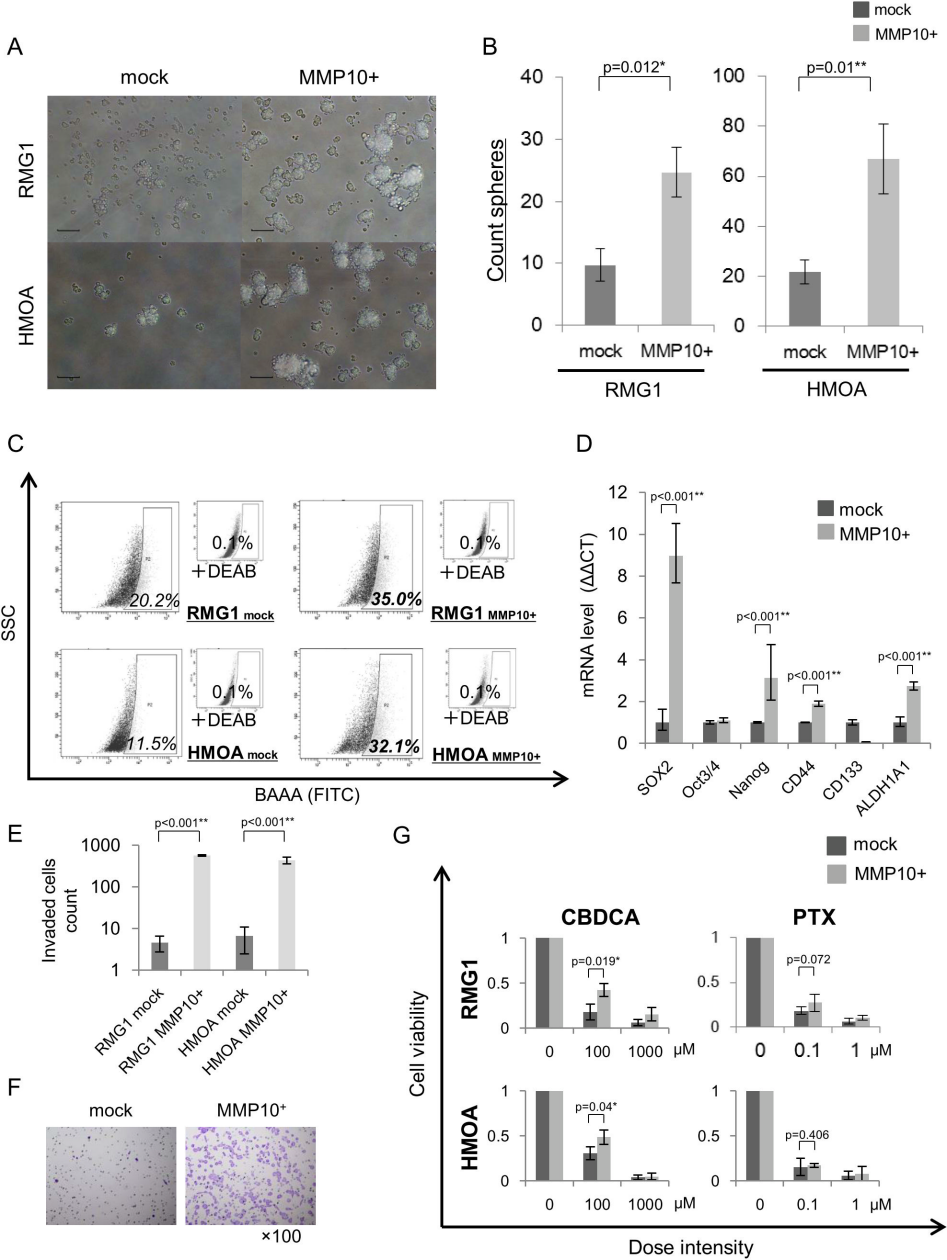
Phenotypes of MMP10-overexpressed cells (**A**) Sphere formation of mock- and MMP10-transfected cells. Seven-day-cultured mock- and MMP10-transfected cells of RMG1 (upper) and HMOA (lower) cell lines under × 100 magnification light microscopic view. Bar scales are 100 μm. (**B**) Sphere-forming assay for mock- and MMP10-transfected cells. Data are shown as means ± SD. (**C**) ALDEFLUOR assay of mock- and MMP10-transfected cell lines. (**D**) Expression of stem cell-related genes in mock- and MMP10-transfected cells. Expression levels of stem cell-related genes were examined by quantitative PCR using the ΔΔCT method. Significantly up-regulated genes in MMP10-transfected cells are shown in the figure. Data are shown as means ± SD. (**E-F**) Matrigel invasion assay for mock- and MMP10-transfected cell lines. Numbers of mock- and MMP10-transfected cells in each of the cell lines, RMG1 and HMOA, are listed in bar graphs in figure E. Data are shown as means ± SD. In figure F, HE-stained membranes of tested columns are shown in ×100 magnification. (**G**) Resistance of mock- and MMP10-transfected cells to chemotherapeutic agents. Calculated cell viability after 48 hrs of treatment with paclitaxel (PTX) and carboplatin (CBDCA) is shown in bar graphs. X-axis is dose intensity of each agent. Y-axis is calculated cell viability from counted number of surviving cells. Data are shown as means ± SD. All statistical analyses for this Figure were performed using bilateral Student's *t* test. *P*-values are shown as follows: *< 0.05, **< 0.01.

**Figure 3:**
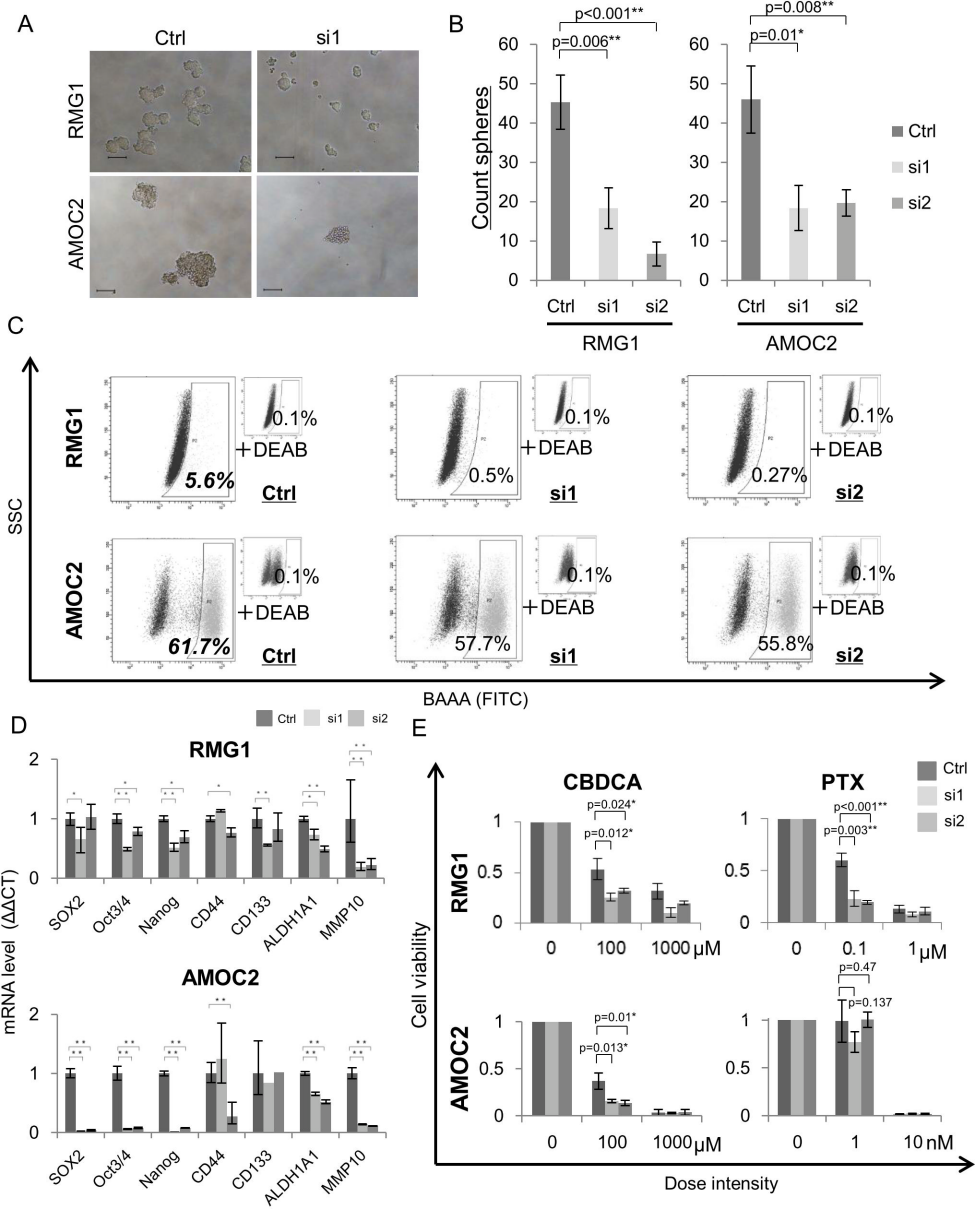
Phenotypes of MMP10 knockdown cells (**A**) Sphere formation of MMP10 knockdown cells. Seven-day-cultured MMP10 knockdown cells of RMG1 (upper) and AMOC2 (lower) cell lines under × 100 magnification light microscopic view. Bar scales are 100 μm. (**B**) Sphere-forming assay of MMP10 knockdown cells. Data are shown as means ± SD. (**C**) ALDEFLUOR assay for MMP10 knockdown cell lines. (**D**) Expression of stem cell-related genes in MMP10 knockdown cells. Expression levels of stem cell-related genes were examined by quantitative PCR using the ΔΔCT method. Data are shown as means ± SD. (**E**) Resistance of mock and MMP10-overexpressed cells to chemotherapeutic. Calculated cell viability after 48 h of treatment with paclitaxel (PTX) and carboplatin (CBDCA) is shown in bar graphs. X-axis is dose intensity of each agent. Y-axis is calculated cell viability from the counted number of surviving cells. Data are shown as means ± SD. All statistical analyses for this Figure were performed using bilateral Student's *t* test. *P*-values are shown as follows: *< 0.05, **< 0.01.

We analyzed the functions of MMP10 in EOC cells by gene overexpression and gene knockdown using siRNAs. Overexpression of MMP10 cDNA was performed by MMP10 cDNA transfection into EOC cells, and the expression was confirmed by qRT-PCR and Western blotting (Figure S3A and S3B). Gene knockdown using siRNAs (siRNA1 and siRNA2) was confirmed by qRT-PCR and Western blotting (Figure S3C and S3D). Sphere forming abilities of RMG1 and HMOA cells were significantly increased by MMP10 overexpression (Figure 2A and 2B). On the other hand, sphere forming abilities of RMG1 and AMOC2 cells were significantly decreased by MMP10 gene knockdown (Figure 3A and 3B). Overexpression of MMP10 increased the ratios of ALDH^+^ cells in RMG1 cells and AMOC cells as shown by the ALDEFLUOR assay (Figure 2C), whereas the ratios of ALDH^+^ cells were decreased by MMP10 gene knockdown (Figure 3C). Expression levels of stem cell-related genes, including *SOX2*, *Oct3/4* (*POU5F1*), *Nanog*, *CD44*, *CD133* (*PROM1*) and *ALDH1A1*, were examined by qRT-PCR. *SOX2*, *Nanog*, *CD44* and *ALDH1A1* were expressed at significantly higher levels in MMP10-overexpressed RMG1 cells than in mock-transfected control cells of RMG1 (Figure 2D). Expression levels of *Oct3/4*, *Nanog* and *ALDH1A1* were significantly decreased in both siRNA1-transfected and siRNA2-transfected RMG1 cells (Figure 3D). *SOX2*, *Oct3/4* and *Nanog* expression levels were decreased by about 100-fold in siRNA1 and siRNA2-transfected AMOC2 cells compared to the levels in control siRNA-transfected AMOC2 cells (Figure 3D). Matrigel invasion assays revealed higher invasion ability of MMP10-overexpressed RMG1 cells and HMOA cells (Figure 2E and 2F). Resistance to chemotherapeutic agents that are commonly used as first-line chemotherapy for ovarian cancer, paclitaxel (PTX) and carboplatin (CBDCA), was examined. MMP10 overexpression increased resistance to CBDCA (Figure 2G), whereas MMP10 knockdown decreased resistance to CBDCA (Figure 3E).

### MMP10 has a role in the tumorigenicity of EOC cells

**Figure 4:**
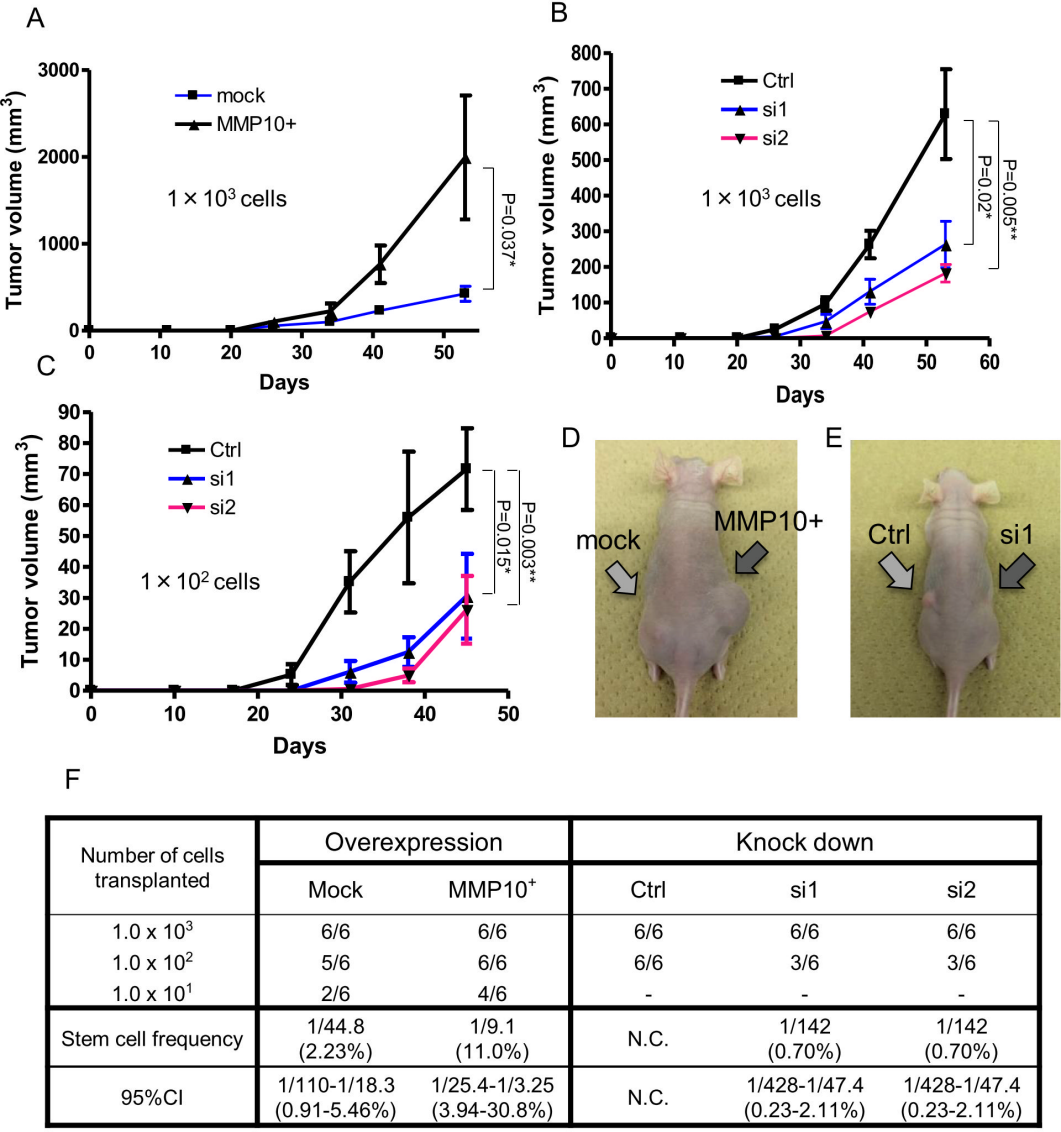
Tumorigenicity *in vivo* xenograft and limiting dilution assay (**A-C**) Tumor growth curves of cells injected into nude mice. Tumor volume in mice in which cells had been injected was checked every week. A: mock and MMP10-overexpressed cells, 1 × 10^3^ cells injected. (B) Ctrl, si1 and si2 cells, 1 × 10^3^ cells injected. (C) Ctrl, si1 and si2 cells, 1 × 10^2^ cells injected. X-axis is the number of days and Y-axis is tumor volume (mm^3^). Data are shown as means ± SD. (**D, E**) Pictures of injected tumors 1 × 103 cells and a mouse. (**F**) Numbers of tumors generated and results of limiting dilution assay. Numbers of tumors generated by the cell lines are shown in table. Stem cell frequencies and 95% confidence intervals were calculated using ELDA as described in experimental procedures. All statistical analyses for this Figure were performed using bilateral Student's *t* test. *P*-values are shown as follows: *< 0.05, **< 0.01.

Since CSCs/CICs were defined by their higher tumor-initiating ability, we investigated the tumor-initiating abilities of MMP10-overexpressed cells and MMP10 knockdown cells using immune-deficient nude mice. We injected MMP10-overexpressed RMG1 cells and MMP10 knockdown RMG1 cells into nude mice. Mock plasmid-transfected control RMG1 cells initiated tumor formation in 6 of 6 mice, 5 of 6 mice and 2 of 6 mice injected with 10^3^ cells, 10^2^ cells and 10^1^ cells, respectively. The ratio of CSCs/CICs in Mock RMG1 cells calculated by ELDA (Extreme Limiting Dilution Analysis: [20]) was 1 in 44.8 cells (2.23%) (Figure 4F). MMP10-overexpressed RMG1 cells initiated tumor formation in 6 of 6 mice, 6 of 6 mice and 4 of 6 mice injected with 10^3^ cells, 10^2^ cells and 10^1^ cells, respectively. The calculated ratio of CSCs/CICs in MMP10-overexpressed RMG1 cells was 1 in 9.8 cells (11.0%) (Figure 4F). The tumor derived from MMP10-overexpressed RMG1 cells showed faster growth than that of the tumor derived from mock-transfected RMG1 cells (Figure 4A and 4D).

Control siRNA-transfected RMG1 cells initiated tumor formation in 6 of 6 mice with both injections of 10^3^ cells and 10^2^ cells. On the other hand, siRNA1 and siRNA2-transfected RMG1 cells initiated tumor formation in 6 of 6 mice and 3 of 6 mice with injection of 10^3^ cells and injection of 10^2^ cells, respectively (Figure 4F). The calculated ratio of siRNA1-transfected and siRNA2-transfected RMG1 cells was 1 in 142 (0.7%) cells (Figure 4F). Furthermore, the tumors derived from siRNA-transfected RMG1 cells showed slower growth than that of tumors derived from control siRNA-transfected RMG1 cells (Figure 4B, 4C and 4E).

### MMP10 enzymatic activity is essential for the maintenance of CSCs/CICs

**Figure 5:**
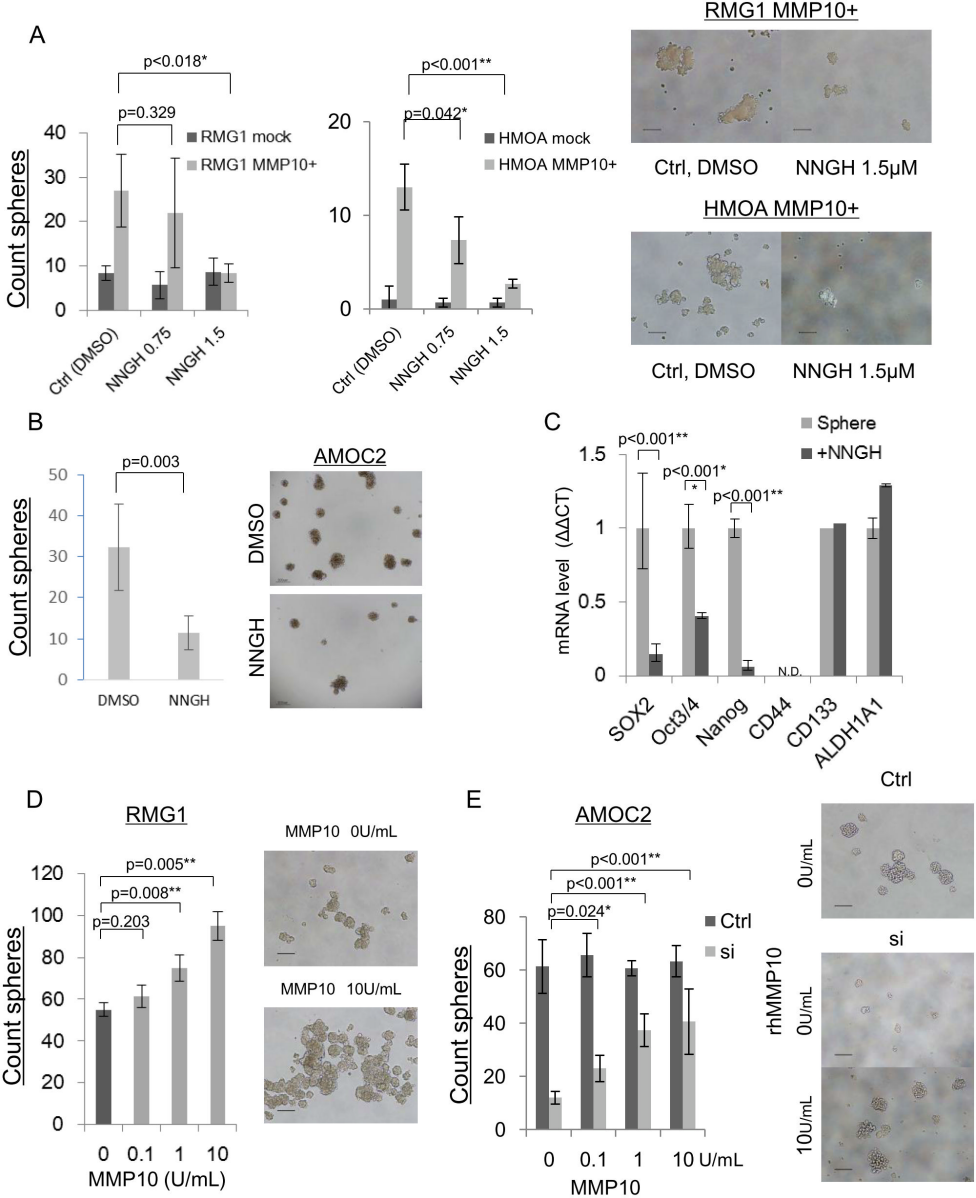
MMP10 inhibition and administration of recombinant human MMP10 protein (**A**) NNGH inhibition of sphere-forming ability in mock and MMP10-overexpressed cells. Number of spheres in each dilution of NNGH (DMSO, 0.75 μM, 1.5 μM) and in each cell line, RMG1 and HMOA. Data are shown as means ± SD. (**B**) NNGH inhibition of sphere-forming ability in AMOC2 cells. Number of spheres in each dilution of NNGH (DMSO, 1.5 μM). Data are shown as means ± SD. The upper pictures are spheres at day 3 after starting culture and the lower pictures are spheres at day 7 in each dilution of NNGH. (**C**) Down-regulation of stem related genes after NNGH administration. Results of quantitative PCR for stem cell-related genes during a period of 6 hours after NNGH administration are shown. The ΔΔCT method was performed using 0 h value as a control. Data are shown as means ± SD. (**D**) Administration of recombinant human MMP10 for RMG1 sphere-forming assay. Counted a number of spheres in each dilution (0, 0.1, 1, 10 U/mL) of recombinant human MMP10. Data are shown as means ± SD. (**E**) Administration of recombinant human MMP10 after MMP10 gene knockdown mediated by siRNA. Number of spheres in each dilution (0, 0.1, 1, 10 U/mL) of recombinant human MMP10-containing medium for MMP10 knockdown cells. Data are shown as means ± SD. All statistical analyses for this figure were performed using bilateral Student's *t* test. *P*-values are shown as follows: *< 0.05, **< 0.01.

MMP10 belongs to the family of matrix metalloproteinases and it has an enzymatic activity. We thus examined whether enzymatic activity is essential for the maintenance of CSCs/CICs in EOC by using an MMP inhibitor, NNGH (N-Isobutyl-N-(4-methoxyphenylsulfonul)-glycylhydroxamic acid). We performed a sphere-forming assay of mock- and MMP10-transfected RMG1 and HMOA cells using media containing NNGH at concentration of 0 μM, 0.75 μM and 1.5 μM. The sphere-forming ability of mock-transfected cells was not affected by NNGH. On the other hand, NNGH significantly inhibited the sphere-forming ability of MMP10-transfected RMG1 and HMOA cells (Figure 5A and 5B). Since AMOC2 cells showed very high internal MMP10 expression (Figure 1E), we performed sphere-forming assay using wild-type AMOC2 cells. NNGH decreased the sphere-forming ability of AMOC2 cells (*P* = 0.003) (Figure 5B). Expression levels of stem cell-related genes were examined using spheres cultured AMOC2 cells in the presence of NNGH. The expression levels of *SOX2*, *Oct3/4* and *Nanog* were decreased by NNGH treatment (Figure 5C).

### Extracellular MMP10 has a role in the maintenance of CSCs/CICs

MMP10 gene overexpression enhanced the CSC/CIC phenotype. MMP10 is a secretory protein, and we examined whether extracellular MMP10 protein has a role in the maintenance of CSCs/CICs using recombinant human MMP10 protein (rhMMP10). The sphere-forming ability of RMG1 cells was increased according to the concentration of hrMMP10 (Figure 5D). MMP10-specific siRNA transfection into AMOC2 cells significantly inhibited the sphere-forming ability (Figure 3A and 3B), and we examined whether hrMMP10 can cancel the effect of siRNA. hrMMP10 significantly increased the sphere-forming ability of siRNA transfected AMOC2 cells, whereas hrMMP10 did not affect the sphere forming ability of control siRNA transfected AMOC2 cells (Figure 5E).

### MMP10 is a marker for poor prognosis and platinum resistance

**Figure 6:**
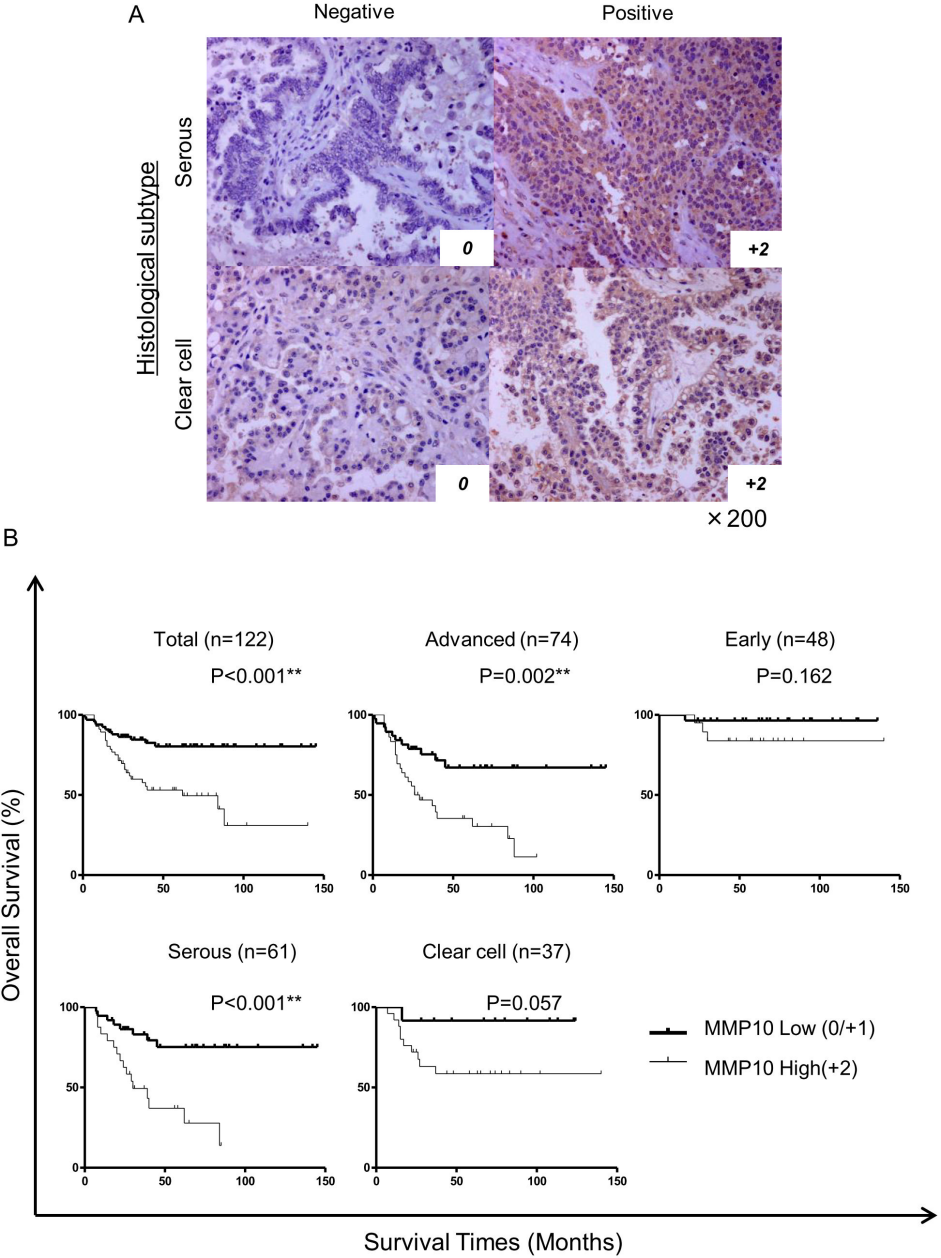
Immunohistochemical staining of clinical samples and statistical analysis (**A**) Immunohistochemically stained ovarian cancer specimens. Clinical specimens stained immunohistochemically using MMP10 antibody and HE for counter staining. All magnifications are ×200. (**B**) Kaplan-Meier curves for overall survival of all cases. The cases were analyzed by expression of MMP10. Total cases: *n* = 122, advanced cases: *n* = 74, early cases: *n* = 48, serous adenocarcinoma cases: *n* = 61 and clear cell adenocarcinoma cases: *n* = 37. Differences in survival curves were analyzed using the log-rank test. *P*-values are shown as follows: *< 0.05, **< 0.01.

To address the significance of the expression of MMP10 in clinical samples, we stained 122 epithelial ovarian cancer specimens immunohistochemically using an anti-MMP10 antibody. The cases were scored as 0, 1+ and 2+ according to the staining intensity. Score 0 and score 1+ were categorized as low expression and score +2 was categorized as high expression (Figure 6A). There were 56 cases in the MMP10 high group and 66 cases in the MMP10 low group. In statistical analysis of clinical characteristics of patients, there were no differences between the MMP10 high and low groups in patient's age, parity, FIGO stage, peritoneal dissemination, lymph node metastasis and achievement of optimal debulking (Table S1). In grouping of histological subtypes, clear cell adenocarcinomas showed a significantly higher MMP10 expression rates (44.6% vs 18.2%; *p* = 0.002). Interestingly, 50 cases with platinum resistance clinically showed a significantly higher rate of MMP10 high expression (80.4% vs 7.58%; *p* < 0.001). In multivariate analysis using a logistic regression model, MMP10 high expression was an independent risk factor of platinum resistance in advanced ovarian cancer (OR, 5.50; 95% CI, 1.67–18.16; *p* = 0.005: Table S2). Kaplan-Meier curves of overall survival of the patients were plotted in several classifications in stages of histological subtypes (Figure 6B). In total cases and advanced cases, there were significant differences in overall survival using log-rank analysis (total cases: *p* < 0.001, advanced cases: *p* = 0.002). Though serous adenocarcinomas showed a significant difference in overall survival (*p* < 0.001), clear cell adenocarcinomas did not reach statistical significance (*p* = 0.057), but MMP10 high cases tended to show poorer prognosis than that of low cases. In multivariate analysis using Cox's proportional hazard model, MMP10 high expression was an independent poor prognostic factor of overall survival in all cases (RR, 2.39; 95% CI, 1.19 – 4.80; *p* = 0.014) and in cases in advanced stage (RR, 2.98; 95% CI, 1.46–6.12; *p* = 0.003: Table 1).

**Table 1:** Multivariate analysis with Cox proportional hazards model for overall survival

Factor	*Total cases (n = 122)*			
	Univariate analysis		Multivariate analysis	
	Risk ratio label (95% confidence interval)	*P*	Risk ratio label (95% confidence interval)	*P*
MMP10 high (score 2)	3.30 (1.67–6.51)	0.001**	2.39 (1.19–4.80)	0.014*
Advanced age (over 50)	1.78 (0.82–3.87)	0.14		
Multipara (≥ 2)	1.78 (0.70–3.24)	0.12		
Histological subtype				
Serous	1.55 (0.82–2.92)	0.17		
Cleacell	0.81 (0.40–1.63)	0.55		
Endometrioid	0.50 (0.16–1.64)	0.26		
Mucinous	0.97 (0.23–4.02)	0.97		
Advanced Stage (over FIGO Stage III)	8.02 (2.85–22.59)	< 0.001**		
Peritoneal dissemination	4.73 (1.93–9.90)	< 0.001**		
Lymph node metastasis	1.48 (0.78–2.81)	0.23		
Platinum resistant	12.46 (5.18–29.96)	< 0.001**	5.75 (2.09–15.81)	0.001**
Nonoptimal debulking surgery	7.00 (3.33–14.74)	< 0.001**	2.84 (1.21–6.64)	0.016*

Factor	*Advanced cases (n = 74)*			
	Univariate analysis		Multivariate analysis	
	Risk ratio label (95% confidence interval)	*P*	Risk ratio label (95% confidence interval)	*P*
MMP10 high (score 2)	2.98 (1.46–6.09)	0.003**	2.98 (1.46–6.12)	0.003**
Advanced age (over 50)	1.38 (0.60–3.16)	0.44		
Multipara (≥ 2)	1.89 (0.83–3.65)	0.14		
Histological subtype				
Serous	0.67 (0.34–1.32)	0.24		
Cleacell	2.01 (0.91–4.43)	0.083		
Endometrioid	0.68 (0.21–2.24)	0.53		
Mucinous	1.81 (0.43–7.65)	0.42		
Advanced Stage (over FIGO Stage III)	–	–		
Peritoneal dissemination	–	–		
Lymph node metastasis	–	–		
Platinum resistant	3.93 (1.62–9.54)	0.001**		
Nonoptimal debulking surgery	4.71 (1.83–12.14)	0.001**	4.70 (1.82–12.13)	0.001**

Factors correlated with deciding FIGO stages were excluded from advanced case's table. The other absent columns are excluded from final model by step wise method (*P < 0.05, **P < 0.01).

### MMP10 activate cannonical Wnt signaling by inhibiting Wnt5a

**Figure 7:**
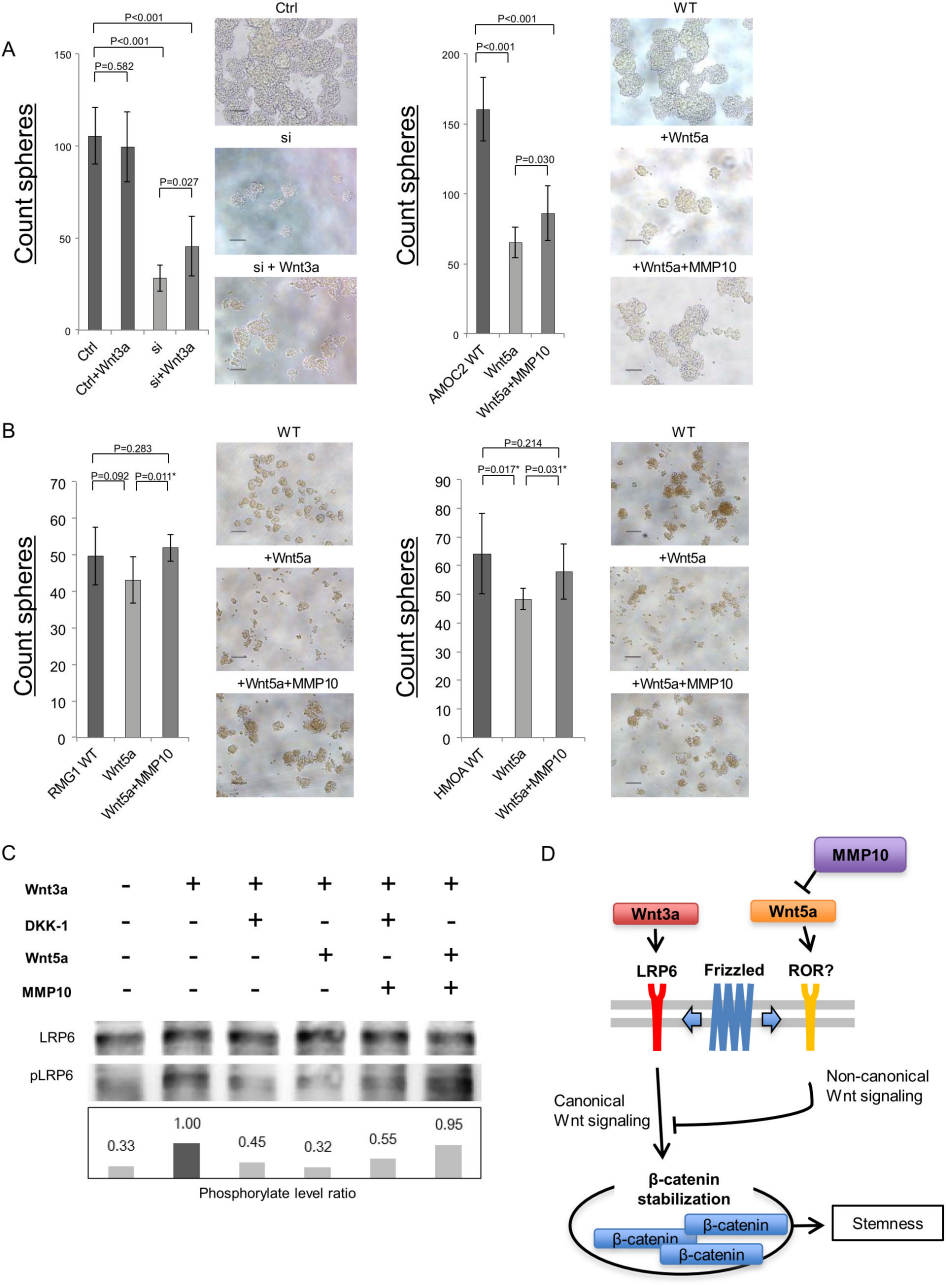
MMP10 activate canonical Wnt signaling by inhibition of noncanonical Wnt signaling ligand Wnt5a (**A**) Caninical Wnt signaling ligand Wnt3a increase the sphere-forming. A canonical Wnt signaling ligand Wnt3a was added to AMOC2 cells that are transfected with control siRNA and MMP10 siRNA. A noncanonical Wnt signaling ligand Wnt5a and MMP10 were added. The numbers of spheres are shown. The numbers of Data are shown as means ± SD. (**B**) MMP10 cancel the inhibition of sphere-formation by Wnt5a. A noncanonical Wnt signaling ligand Wnt5a and MMP10 were added. The numbers of spheres are shown. The numbers of Data are shown as means ± SD. (**C**) A Western blot of canonical signaling. Total LRP6 protein and phosphorylated LRP6 (pLRP6) were detected by a Western blot. The intensity of bands were analyzed by imageJ software, and the phosphorylation levels of LRP6 were calculated as: intensity of phosphorylated LRP6/intensity of total LRP6. AMOC2 cells were incubated in medium containing Wnt3a, DKK1, Wnt5a and MMP10. (**D**) Schematic model of MMP10 in ovarian CSCs/CICs. A canonical Wnt ligand Wnt3a activate canonical Wnt signaling by activation of LRP6. A noncanonical Wnt lignad Wnt5a inhibit canonical Wnt signaling through an unknown receptor. MMP10 inhibit Wnt5a and increase the canonical Wnt signaling and following activation of β-Catenin that induce transcriptions of stem cell-related genes including SOX2, Oct3/4 and Nanog.

To explore the molecular mechanisms how extracellular MMP10 increase the stemness, we analyzed the Wnt signaling that is known as stem cell-related signaling pathway [21]. A recent study revealed that MMP3 activate canonical Wnt signaling by inhibition of noncanonical Wnt signaling ligand, Wnt5b [22]. A previous study revealed that a Wnt5b orthologue Wnt5a suppress ovarian cancer cells [23], and our data revealed that Wnt5a showed relative higher expression in ovarian cancer cells than that of Wnt5b (data not shown). We therefore analyzed the functions of canonical Wnt signaling ligand Wnt3a and noncanonical Wnt signaling ligand Wnt5a. Wnt3a significantly increased the sphere-forming ability of AMOC2 cells that are transfected with MMP10 siRNA (Figure 7A). On the other hand, Wnt5a significantly decreased the sphere-forming ability of AMOC2 cells (Figure 7A). The decrease of sphere-forming ability by Wnt5a was partially cancelled by MMP10 (Figure 7A). This results suggest that activation of canonical Wnt signaling has a role in sphere-forming and activation of noncanonical Wnt signaling inhibit sphere-forming. To generalize these observation, we performed experiments using additional two cell lines RMG1 and HMOA. The sphere-forming were inhibited by Wnt5a, and it could be cancelled by MMP10 (Figure 7B).

The activation of canonical Wnt signaling was addressed by a Western blot. Wnt3a activate canonical Wnt signaling by phosphorylation of LRP6 that was inhibited by a Wnt signaling inhibitor DKK1. Wnt5a inhibited canonical Wnt signaling. MMP10 canceled the inhibition by Wnt5a; however, DKK1 could not cancel (Figure 7C). These results indicate that MMP10 activate canonical Wnt signaling by inhibiting noncanonical Wnt signaling ligand Wnt5a (Figure 7D).

## DISCUSSION

In this study, we identified MMP10 as a CSC/CIC-related gene in EOC. MMP10 is a member of the matrix metalloproteinase family, classified as stromelysins [24]. MMP10 cleaves various types of extracellular matrix and activates other MMPs including MMP-1,7, 8 and 9 [25]. MMP10 is expressed in various types of cancers including esophageal cancer [26], lymphoma [27], head and neck cancer [28, 29] and lung cancer [30]. Expression of MMP10 was reported to be associated with poor prognosis and its function was reported to have a role in tumor development. MMP10 was reported to have a role in the maintenance of mice lung cancer stem cells [31]; however, its function in human CSCs/CICs of EOC is still elusive. We showed that overexpression of MMP10 increased the ratios of CSCs/CICs as revealed by the ALDEFLUOR assay, enhanced tumorigenicity in mice, and increased sphere-forming ability and resistance to chemotherapeutic agents. The reverse phenomena were observed by MMP10 knockdown. MMP10 is known as a proteinase of the extracellular matrix, and an MMP inhibitor, NNGH, inhibited the stemness of EOC cells. Addition of recombinant human MMP10 to the culture medium of MMP10 knockdown cells resulted in partial recovery of sphere-forming ability. These results indicate that MMP10 has a role in the maintenance of CSCs/CICs in EOC, that protease activity of MMP10 is important and that extracellular MMP10 might be sufficient for EOC cell stemness.

MMPs regulate a variety of physiological processes and signaling events, and they are thus represent key players in molecular communication between a tumor and stroma [32]. MMP10 digests the extracellular matrix, including collagen types III–V and fibronectin [33], but other substrates have been reported recently. Screening of substrates of MMP10 by TAILS (Terminal Amine Isotopic Labeling of Substrates) workflow revealed that MMP10 cleaves ADAMTSL1 and PDGFRα [34]. We thus examined ADAMTSL1 gene expression in ovarian cancer cell lines in an adherent and sphere condition, but the expression was not detectable (data not shown). We then examined the expression of PDGFRα and its level of phosphorylation in CSC/CIC and non-CSC/CIC conditions. Spheres showed lower PDGFRα gene expression than that in adherent cultured cells, and phosphorylation and cleavage of PDGFRα were not observed in MMP10-overexpressed cells (data not shown).

In this study, we identified that MMP10 activate canonical Wnt signaling. A previous study revealed that MMP3 a highly homologous protein as MMP10, has a role in the regulation of mouse mammary stem cell, and growht of the mammary epithelial cells *in vivo* [22]. They found that MMP3 inhibit noncanonical Wnt signaling ligand Wnt5b. Interestingly, only hemopexin (HPX) domain can associate with Wnt5b and only HPX domain is sufficient for the regulation of mammary stem cells. HPX domain of MMP families has a similar structure as heme, and it has functions as cell migration [35], indicating that HPX domain might activate some signaling. In this study, we observed that NNGH is sufficient to inhibit stemness of ovarian CSCs/CICs. Since NNGH inhibit the enzyme activity of MMPs [36], our result indicate that enzymatic activity of MMP10 is necessary. However, NNGH is a cell membrane permeable substance, and there are still possibilities that NNGH might inhibit the secretion of MMP10 or inhibit the interaction of MMP10 and Wnt5a. Thus further detailed analysis is needed to be concluded. Our results showed that suppression of MMP10 by siRNAs or NNGH decreased the expressions of stem cell-related genes including SOX2, Oct3/4 and Nanog. Since activation of Wnt signaling increase the transcriptions of SOX2, Oct3/4 and Nanog [37, 38], the canonical Wnt signaling might be the major mechanisms of the relation of MMP10 and stemness of ovarian CSCs/CICs.

Although the exact mechanism of stemness regulation is still elusive, MMP10 might be a suitable target molecule for CSC/CIC-targeting therapy. A high expression level of MMP10, as revealed by immunohistochemical staining, was shown to be an independent poor prognostic factor by multivariate analysis using Cox's proportional hazards model, a high expression level of MMP10 was shown to be an independent risk factor of platinum resistance in logistic multivariate analysis. Results of *in vitro* assays using MMP10-overexpressed cells and MMP10 knockdown cells support the data obtained by immunohistochemistry. Therefore, inhibition of MMP10 expression may improve the reactivity to chemotherapy. A previous study showed that MMP10 was expressed at higher levels in cisplatin-resistant sublines, CP70 and CP200, than in the parental ovarian cancer cell line A2780 [39]. Therefore, targeting MMP10 might improve the sensitivity to chemotherapy, and the combination of MMP10-targeting therapy and chemotherapy might be a reasonable approach for EOC. MMP10 is generally expressed in ovarian cancer cells, but it is barely expressed in normal organs except for the uterus. In standard surgical treatment of ovarian cancer, the uterus is removed. Thus, MMP10-targeting therapy as adjuvant therapy after primary surgery might not show any adverse effects. Furthermore, no lethal events in embryonic and postnatal stages were observed in MMP10 knockout mice, indicating the safety of targeting MMP10 [40]. However, a function of MMP10 in repair of injured tissue has also been reported, and MMP10 is expressed in keratinocytes of wounded epithelial skin and its expression level must be tightly controlled for appropriate matrix contact [41]. A muscle ischemia model revealed that the expression of chemokines including cxcl1 is regulated by MMP10 and that MMP10 is needed for muscle regeneration and repair [42]. Therefore, inhibition of MMP10 activity may affect surgical wound repair.

MMPs have been thought to be promising targets of cancers for several decades. Clinical trials using a synthetic metalloproteinase inhibitor have been performed, but the results were disappointing [43]. Some clinical trials have been performed for ovarian cancer. In a phase III RCT of Tanomastat (BAY12-9566), an MMP-2, -3 and -9 inhibitor, there was no evidence of an impact on PFS or OS [44]. A phase I study of Marimastat (MMP-1, 2, 7, 9 and 14 inhibitor) plus carboplatin was performed in patients with ovarian cancer who previously responded to a platinum-based regimen, and the drug was tolerated well (Thomas, H 1997). However, the phase III study revealed that there was no difference in response rate. In this study, we showed that there is a relationship between MMP10 expression and resistance to chemotherapy. Thus, combination of MMP10-targeting therapy and chemotherapy might be effective.

In summary, our results indicate that MMP10 is expressed in CSCs/CICs of EOC and has an essential role in the maintenance of CSCs/CICs. MMP10 is a promising target of CSC/CIC-targeting therapy for EOC, especially in chemotherapy-resistant cases. A combination of MMP10-targeting therapy and chemotherapy might improve the prognosis of EOC cases.

## MATERIALS AND METHODS

### Cell lines and culture conditions

In this study, we used three cell lines, RMG1 (clear cell adenocarcinoma), HMOA (endometrioid adenocarcinoma) and AMOC2 (serous adenocarcinoma). RMG1 cells were cultured in DMEM/F12 (Life Technologies, Grand Island, NY, USA). HMOA and AMOC2 cells were cultured in RPMI1640 (Sigma-Aldrich, St Louis, MO, USA). Each medium contained 10% fetal bovine serum (FBS) and was incubated in a humidified 5% CO_2_ incubator at 37°C.

### ALDEFLUOR assay and isolation by FACS

The ALDEFLUOR assay (Stem Cell Technologies^™^, Vancouver, BC, Canada) was performed to determine ALDH^+^ cells according to the manufacturer's protocol. Cells were counted and suspended in assay buffer containing 1 μM per 1 × 10^6^ cells of the ALDH substrate, boron-dipyrrometheneaminoacetaldehyde (BAAA), and incubated for 50 min at 37°C. Each sample was treated with 50 nM of an ALDH-specific inhibitor, diethylaminobenzalydehyde (DEAB), as a negative control. BAAA-stained cells were analyzed and sorted using BD FACSAria^™^ II (BD Biosciences, San Jose, CA, USA).

### Total RNA isolation and microarray preparation

Total RNA of ALDH^+^ cells and ALDH^-^ cells derived from AMOC2 cells were isolated from collected cells using an RNeasy Mini Kit (QIAGEN, Valencia, CA) following the manufacturer's protocol. We used the commercially available Low Input Quick Amp Labeling Kit (Agilent Technologies). Purified total RNA (3 μg) was reverse-transcribed to generate double-stranded cDNA using an oligo dT T7 promoter primer and reverse transcriptase. Then cRNA was synthesized using T7 RNA polymerase, which simultaneously incorporated Cy3- or Cy5-labeled cytidine triphosphate. During this process, the samples of RNA derived from ALDH^+^ cells were labeled with Cy5, whereas the RNA derived from ALDH^-^ cells were labeled with Cy3 as control cells. Quality of the cRNA was checked using the Nano Drop. Cy3-labeled cRNA derived from ALDH^+^ cells and Cy5-labeled cRNA derived from ALDH^-^ cells were combined and then fragmented using a gene expression hybridization kit (Agilent Technologies). The cRNAs were hybridized to a 60-mer probe oligonucleotide microarray (G4845A human GE 4 × 44 k V2 Microarray kit) and incubated for 20 hours at 50°C. The fluorescent intensities were determined by an Agilent Technologies Scanner G2505C. Samples of ALDH^+^ cells were labeled with Cy3, whereas ALDH^-^ cells were labeled with Cy5. Microarray raw data and processed data have been deposited in the NCBI GEO database (GSE64539).

### Primary ascites cell culture

Primary ovarian cancer cells were collected from the patient's ascites. Detailed procedures are described in Supplemental Experimental Procedures. Written informed consent was obtained from the patient before collecting the ascites.

### Generation of a stable cell line overexpressing MMP10

The retrovirus vectors used in this study, the procedure for preparing retroviral vectors, and transduction are described in Supplemental Experimental Procedures.

### MMP10 gene knockdown mediated by small interference RNA

Procedure for the MMP10 gene knockdown experiment procedures are described in detail in Supplemental Experimental Procedures.

### Sphere-forming assay

Cells were cultured in 6-well ultralow attachment surface dishes (Corning Inc., Corning, NY, USA) at 3,000 cells per well for RMG1 and HMOA and at 1,000 cells per well for AMOC2, because formation of countable spheres was difficult with RMG1 and HMOA at 1,000 cells per well. The number of spheres was counted only spheres which diameter was over 50 μM after 7 days of cell culture, under light microscopy. The cells were cultured in serum-free DMEM/F12 (Invitrogen) medium supplemented with 20 ng/ml recombinant human epithelial growth factor (Life Technologies) and 10 ng/ml human basic fibroblast growth factor (Sigma-Aldrich).

Wnt3a, Wnt5a and MMP10 (R and D systems) were added at concentrations of 200 ng/mL, 1000 ng/mL and 10U, respectively.

### Matrigel invasion assay

The procedures are described in detail in Supplemental Experimental Procedures.

### Resistance to chemotherapeutic agents

Cell counting and calculation of the ratio of surviving cells were performed. Procedures are described in detail in Supplemental Experimental Procedures.

### RT-PCR, qRT-PCR and immunoblotting

Detailed protocols are provided in Supplemental Experimental Procedures.

### *In vivo* tumorigenicity and limiting dilution assay

Cells (mock, MMP10-overexpressed, si ctrl, si1 and si2-transfected cells) were injected into nude mice, and the mice were observed weekly. Procedures are described in detail in Supplemental Experimental Procedures.

### MMP inhibition assay using NNGH

MMP inhibition was performed using NNGH (N-Isobutyl-N-(4-methoxyphenylsufonyl)-glycylhydroxamic acid), an inhibitor of several members of the MMP family including MMP10. Procedures are described in detail in Supplemental Experimental Procedures.

### Recombinant human MMP10 administration assay

RMG1 and MMP10 knockdown AMOC2 cells were prepared. Recombinant human MMP10 (910-MP; R & D Systems) diluted in a medium conditioned for the sphere-forming assay was used for the cells. Recombinant MMP10 was added to media at 10 U/ml, 1 U/ml and 0.1 U/ml. After 7 days of culture, the number of spheres was counted as described previously.

### Patients and specimens; IHC staining

Surgical specimens used for immunohistochemically staining were obtained from 122 patients with primary epithelial ovarian cancer who had been treated at Sapporo Medical University Hospital during the period from 2001 to 2011. A control sample for the endometrium was also obtained from a patient in whom the cancer was restricted to the ovary with no invasion. Written informed consent was obtained from each patient according to the guidelines of the Declaration of Helsinki. Sections (5 μm in thickness) of formalin-fixed paraffin-embedded tumors were immunostained a using monoclonal antibody of MMP10 (R & D; Clone 110304). Procedures are described in detail in Supplemental Experimental Procedures.

### Quantification of MMP10 staining

Immune reactivity levels for MMP10 were categorized as 0, +1 and +2 by the stained area and density of the cancer lesion. A score of zero was given for a cancer lesion that was not stained. A score of +1 was given for a partially defected stained area in cancer lesion or stained weakly. A score of +2 was given for cases in which almost all of the lesion (> 90%) was stained. Finally, we classified them into two groups: scores of 0 and +1 as an MMP10 low group and a score of +2 as a high group. This grouping was evaluated by a gynecologist and pathologists.

### Western blot

A Western blot was performed as described previously [45]. AMOC2 cells were starved in FBS free medium for 12 hours, then added Wnt3a, DKK1, Wnt5a and MMP10 at concentrations 40 ng/mL, 500 ng/mL, 100 ng/mL and 10 U, respectively and incubated for 2 hours. The cells were lysed by an SDS sample buffer. Protein samples were applied to SDS-PAGE, and separated proteins were transferred onto a PVDF membrane (Immobion-P transfer membrane, Melck). After blocking with 5% skim milk in Tris-buffered saline containing 0.03% Tween20 (TBS-T) for 40 min, the membrane was incubated with a primary antibody at a dilution of 1:1000 in TBS-T containing 5% skim milk for 40 min at room temperature. Anti-LRP6 (Cell Signaling) and anti-phosphorylated LRP6 (Cell Signaling) antibodies were used.

### Statistical analysis

Statistical analyses were performed with SPSS (version 21 for Windows; SPSS Inc), and GraphPad Prism (version 4.0 for Windows; GraphPad Software Inc) was used for plotting Kaplan-Meier curves. Procedures are described in detail in Supplemental Experimental Procedures. In all analyses, *P*-values < 0.05 were considered as statistically significant and shown as * < 0.05, ** < 0.01.

## SUPPLEMENTARY MATERIALS FIGURES AND TABLES


