# Supplementary material for: Matrix metalloproteinase-10 regulates stemness of ovarian cancer stem-like cells by activation of canonical Wnt signaling and can be a target of chemotherapy-resistant ovarian cancer
Source: Oncotarget. 2016 Apr 8;7(18):26806–22. doi: 10.18632/oncotarget.8645 (PMC5042016; doi:10.18632/oncotarget.8645)
Supplement: Supplementary file 1 [file oncotarget-07-26806-s001.pdf]

Matrix metalloproteinase-10 regulates stemness of ovarian cancer stem-like cells by activation of canonical Wnt signaling and can be a target of chemotherapy-resistant ovarian cancer

Supplementary Materials

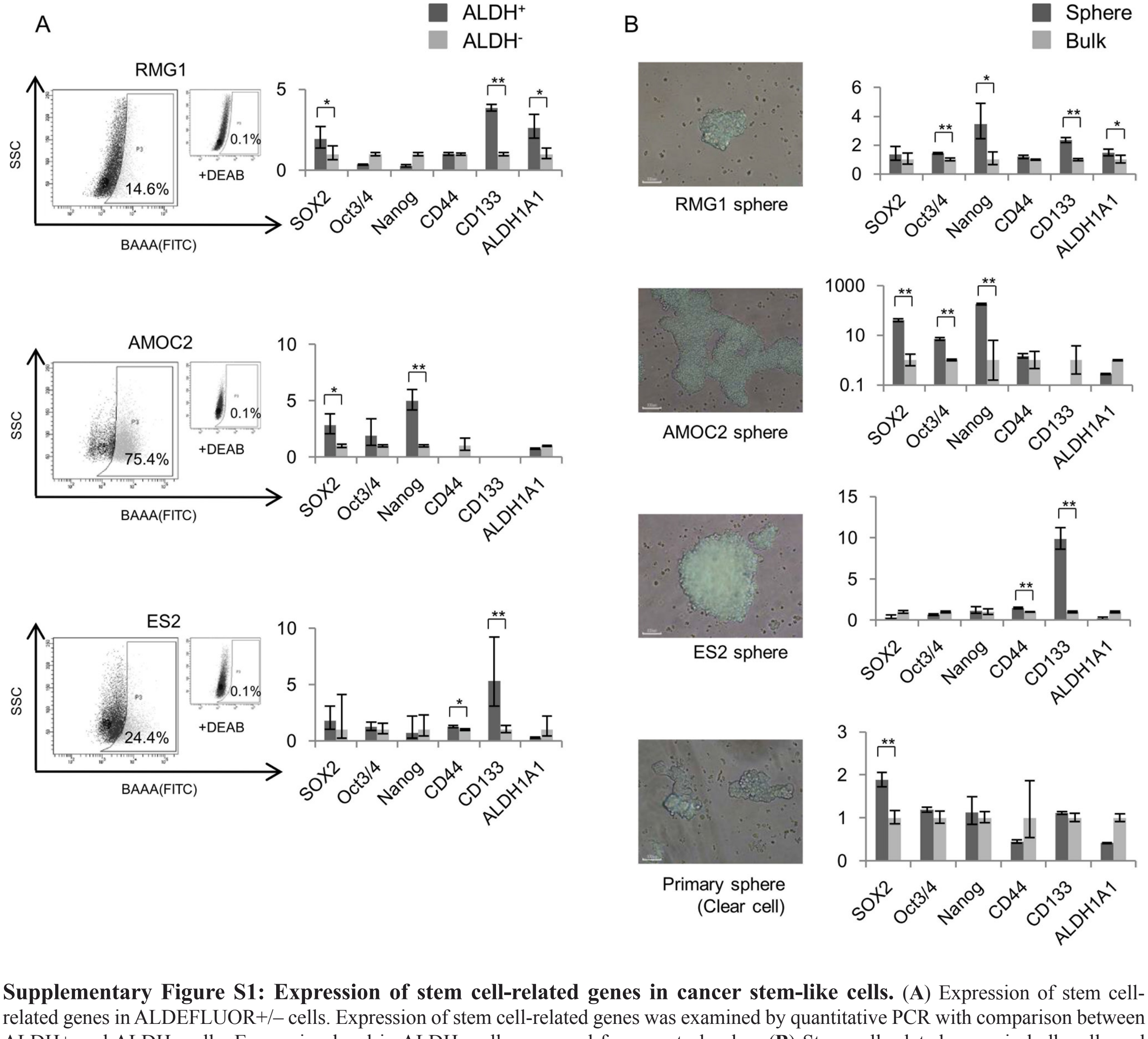

Supplementary Figure S1: Expression of stem cell-related genes in cancer stem-like cells. (**A**) Expression of stem cell-related genes in ALDEFLUOR+/– cells. Expression of stem cell-related genes was examined by quantitative PCR with comparison between ALDH+ and ALDH- cells. Expression level in ALDH- cells was used for a control value. (**B**) Stem cell-related genes in bulk cells and spheres. Stem cell-related genes were examined by quantitative PCR with comparison between adherent cultured cells (bulk) and spheres. In significantly up-regulated genes in cancer stem-like cells, ALDH+ cells and spheres are shown in figures A and B. Data are shown as means ± SD. All statistical analyses for this Figure were performed using bilateral Student's *t* test. *P*-values are shown as follows: *< 0.05, **< 0.01.

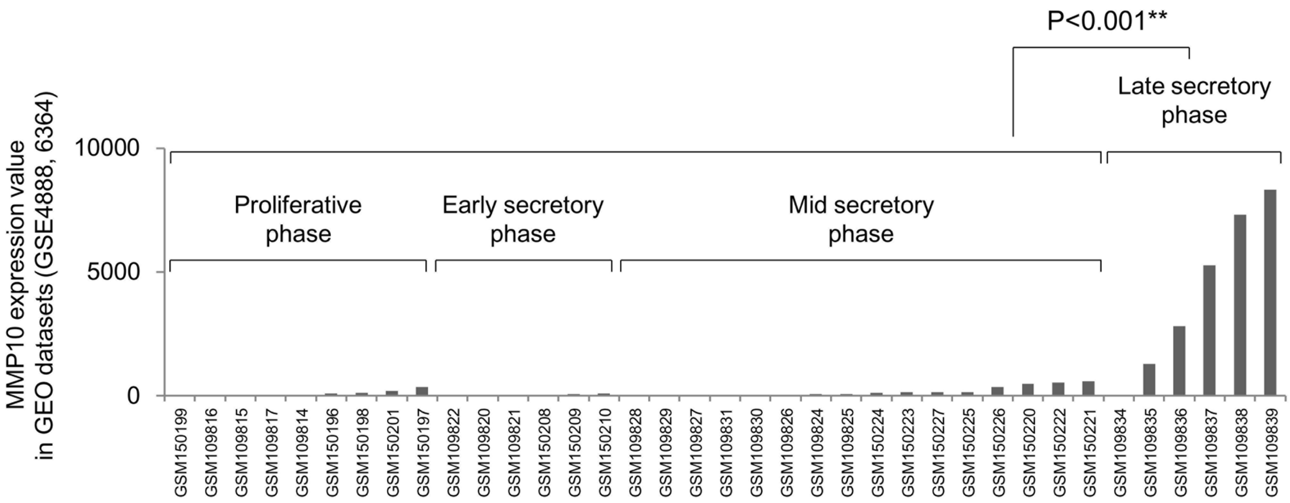

Supplementary Figure S2: MMP10 gene expression in the endometrium during the menstrual cycle. MMP10 mRNA expression levels in each dataset are shown in a bar graph. Datasets of a microarray in each menstrual phase were collected from the NCBI GEO database, as described in supplemental experimental procedure. Statistical analysis was performed using bilateral Student's *t* test. *P*-values are shown as follows: *< 0.05, **< 0.01.

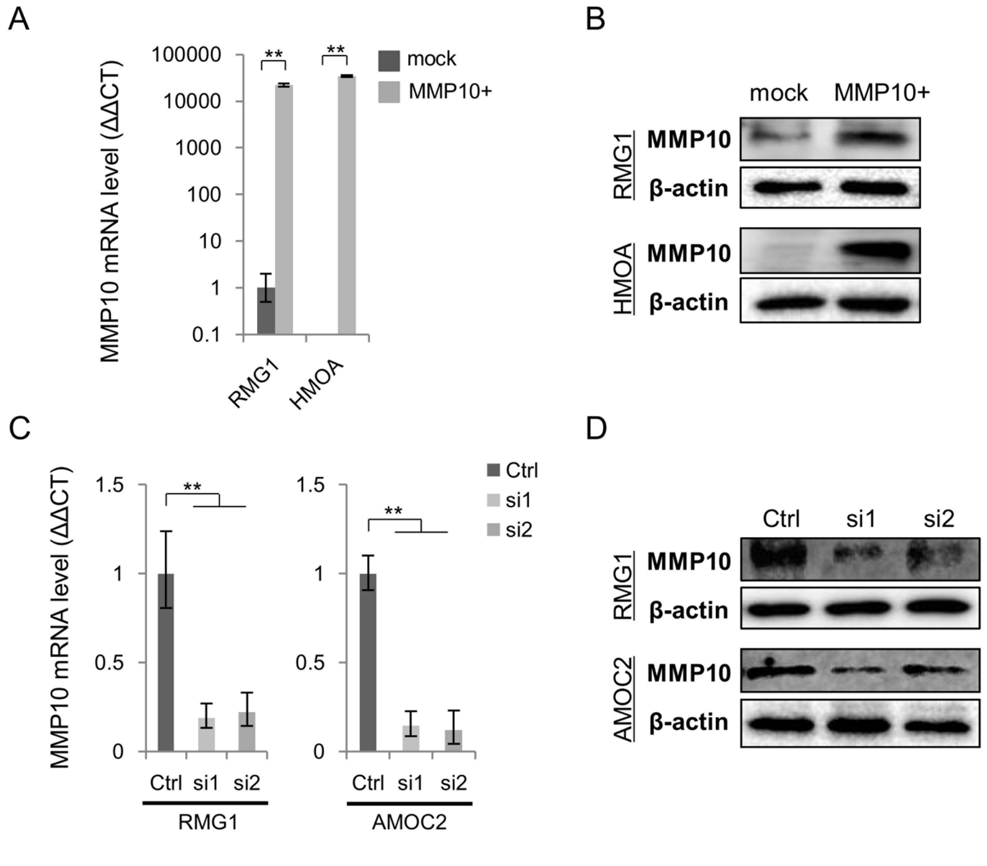

Supplementary Figure S3: MMP10 expression levels in MMP10-overexpressed cells and MMP10 knockdown cells. (**A**) Quantitative PCR for MMP10-overexpressed cells. MMP10 gene expression levels were examined by quantitative PCR in mock and MMP10-overexpressed cells of RMG1 and HMOA. Data are shown as means ± SD. (**B**) Western blotting for MMP10-overexpressed cells. MMP10 protein expression levels in mock and MMP10-overexpressed cells were examined using Western blotting. Expression of β-actin was used as an internal control. (**C**) Quantitative PCR for MMP10 knockdown cells. MMP10 gene expression levels in knock down cells (ctrl, si1 and si2-transfected cells) of RMG1 and AMOC2 cells were examined by quantitative PCR. Data are shown as means ± SD. (**D**) Western blotting for MMP10 knockdown cells. MMP10 protein expression levels in knockdown cells (ctrl, si 1 and si2-transfected cells) of RMG1 and AMOC2 were examined using Western blotting. Expression of (β-actin was used as an internal control. All statistical analyses for this Figure were performed using bilateral Student's *t* test. *P*-values are shown as follows: *< 0.05, **< 0.01.

Supplementary Table S1: Clinical characteristics of patients

**Characteristic**		**MMP10 expression**			**Total**
		**High** (***N*** **= 56)**	**Low** (***N*** **= 66)**	***P***	**(*N* = 122)**
Age	Mean ± SD	55.1 ± 10.6	55.1 ± 10.7	0.40	55.1 ± 10.2
	Range	32–81	29–81		29–81
Parity (%)	0	14 (25)	25 (37.9)	0.54	39 (32)
	1	15 (26.8)	11 (16.7)		26 (21.3)
	2	19 (34.0)	21 (31.8)		40 (32.8)
	3	7 (12.5)	8 (12.1)		15 (12.3)
	4	1 (1.79)	1 (1.52)		2 (1.64)
Histological subtype (%)	Serous	24 (42.9)	37 (56.1)	0.15	61 (50)
	Clearcell	25 (44.6)**	12 (18.2)	0.002**	37 (30.3)
	Endometrioid	5 (8.93)	13 (19.7)	0.10	18 (14.8)
	Mucinous	2 (3.57)	4 (6.06)	0.53	6 (4.92)
FIGO Stage No. (%)	I	18 (32.1)	24 (36.4)	0.86	42 (34.4)
	II	2 (3.57)	4 (6.06)		6 (4.92)
	III	32 (57.1)	34 (51.5)		66 (54.1)
	IV	4 (7.14)	4 (6.06)		8 (6.56)
Peritoneal dissemination (%)		31 (55.4)	40 (60.6)	0.56	71 (58.2)
Lymph node metastasis (%)		20 (35.7)	16 (24.2)	0.17	36 (30)
Optimal debulking surgery (%)		31 (55.4)	41 (62.1)	0.45	72 (59)
Platinum resistant (%)		45 (80.4)**	5 (7.58)	< 0.001**	50 (41)

*$P < 0.05$, **$P < 0.01$.

Supplementary Table S2: Multivariate analysis using logistic regression model for risk of platinum resistance

Factor		Univariate OR (95% CI)	*P*	Multivariate OR (95% CI)	*P*
MMP10 high		3.48 (1.51–8.05)	0.004**	5.50 (1.67–18.16)	0.005**
Advanced age (> 50)		1.80 (0.57–5.67)	0.32		
Multipara (≥ 2)		2.02 (0.73–5.58)	0.17		
Histological subtype					
	Serous	0.81 (0.26–2.48)	0.71		
	Clearcell	N.C.	-		
	Endometrioid	0.20 (0.04–0.88)	0.034*	0.21 (0.04–1.04)	0.055
	Mucinous	N.C.	-		

In clear cell and mucinous adenocarcinomas, advanced cases which administered chemotherapies were all platinum resistant, and OR are not calculated (N.C.). *< 0.05, **< 0.01.

Supplementary Table S3: Firstline chemotherapy regimens

Regimen		Total
TC (PTX/CBDCA)		69
DC (DTX/CBDCA)		22
CPT/CDDP		3
Others		
	CPT/NDP	1
	Gem/P	1
	DP (DTX/CDDP)	1
No adjuvant chemotherapy		25

MATERIALS AND METHODS

MMP10 mRNA expression in the menstrual cycle

As briefly described in the article, we collected datasets of a cDNA microarray from the NCBI GEO database. GSE4888 and GSE6364 were used for analysis. We checked the expression level of MMP10 from these datasets and performed analysis using in Student's *t*-test.

Primary ascites cell culture

Primary ovarian cancer cells were collected from the patient's ascites. The diagnosis of the resected primary lesion was mixed cancer, clear cell adenocarcinoma and borderline mucinous adenocarcinoma. After 3 kinds of chemotherapies had been administered, cancer progressed and cancerous ascites filled the patient's abdominal cavity. We collected ascites in a sterilized condition and cultured the cells extracted from ascites in DMEM/F12 (Invitrogen) medium containing 10% FBS. At the same time, sphere culturing was performed using an ultra low attachment flask (Corning Inc., Corning, NY, USA) in serum-free DMEM/F12 medium supplemented with 20 ng/ml recombinant human epithelial growth factor (Life Technologies) and 10 ng/ml human basic fibroblast growth factor (Sigma-Aldrich). Written informed consent was obtained from the patient before collecting the ascites.

Generation of a stable cell line overexpressing MMP10

Transduction of MMP10 DNA into RMG1 and HMOA cells was performed by a retrovirus-mediated method. MMP10 cDNA cloned from RMG1 sphere cDNA by RT-PCR was inserted into the pMXs-puro retrovirus vector. Primer pairs used for cloning MMP10 were 5′- CGCGGATCCGCCACCATGATGCATCTTG CATTCCT-3′ (with BamH1) and 5′- CCCTCGAGCTAG CAATGTAACCAGCTGT-3′ (with Xho1). Retrovirus infection was performed as described previously (Morita, 2000). Two days after infection, 1 mg/ml puromycin was added to the medium to select a stably transduced subline. The expression of MMP10 was confirmed by qPCR and Western blots (Figure S3A).

MMP10 gene knockdown mediated by small interference RNA

An MMP10 gene knockdown experiment was performed using small interfering RNA (siRNA). MMP10 siRNA (NM 002425) and negative control siRNA were purchased from ORIGENE (Origene Technologies, ID4319 Trilencer-27 Human siRNA, Rockville, USA). RMG1 and AMOC2 cells were cultured in a 10 cm dish, and transfections were carried out using Lipofectamine RNAi max (Life Technologies) in Opti-MEM according to the manufacturer's instructions. We used these cells after 48 hours of incubation for detecting mRNA and after 72 hours of incubation for sphere forming assay, examination for resistance to chemotherapeutic agents, ALDEFLUOR assay, xenografting and collecting cell lysates for immunoblotting.

Matrigel invasion assay

Invasion ability of mock and MMP10-overexpressed cells was evaluated using Matrigel invasion chambers (BD1™ Biosciences Discovery Labware). The cells were counted and resuspended at 1×10^4 cells and then plated in each upper chamber in serum-free DMEM. The outer chambers were filled with DMEM containing 10% FBS. Cells were incubated for 48 hours, and invading cells were stained with hematoxylin, mounted on slides, and counted in a × 100 field by light microscopy.

Resistance to chemotherapeutic agents

Cells were counted and 5×10^5 cells were cultured in a medium with 10% FBS overnight. After overnight incubation, the medium was changed to a medium containing CBDCA and PTX at several concentrations. CBDCA was diluted into 100 and 1000 μM and PTX was diluted into 0.1 and 1 μM. Only AMOC2 cells were extremely sensitive to PTX, and their concentrations were therefore set to 1 and 10 nM. Cells cultured in an agent-free medium were prepared as control cells. After 48 hours of incubation in the medium with agents, all cells are collected gently using a cell scraper and stained by trypan blue. Cells that were not stained were counted as surviving cells, and cell survival rates were calculated from the ratio of treated/control cells.

Reverse transcription polymerase chain reaction analysis (RT-PCR)

We performed RT-PCR for examination of MMP10 mRNA expression in normal organs. A cDNA panel for a set of normal human adult tissues was purchased (Clontech), and only cDNA of the uterus was purchased from Wako (Wako, Japan). The thermal cycling conditions were 94 for 2 min, followed by 40 cycles of 15 sec at 94°C, 30 sec at 60°C, and 30 sec at 72°C. Primer pairs used for MMP10 were 5′- GGCTCTTTCACTCAGCCAAC-3′ and 5′- TCCCGAAGGAACAGATTTTG-3′. Primers for G3PDH (glyceraldehyde-3-phosphate dehydrogenase), used as an internal control, were 5′- ACCACAGTCCATGCCATCAC-3′ and 5′-TCCACCACCCTGTTGCTGTA-3′.

Quantitative real-time PCR analysis (qPCR)

Quantitative real-time PCR was performed using an ABI PRISM 7000 Sequence Detection System (Applied Biosystems, Foster City, CA) according to the manufacturer's protocol. We used probes for detection that were designed by the manufacturer (TaqMan Gene

expression assays; Applied Biosystems). Probes were for SOX2, Nanog, POU5F1, CD44, PROM1, ALDH1A1 and MMP10. Thermal cycling was 40 cycles of 95°C for 15 seconds followed by 60 for 1 min. GAPDH was used as internal control.

Immunoblotting analysis

Cells were lysed with ice-cold Nonidet P-40 (NP40) lysis buffer, incubated on ice for 10 min, mixed with 2 × sample buffer, and boiled in 100°C for 5 min. Samples were separated by sodium dodecyl sulfate-polyacrylamide gel electrophoresis (SDS-PAGE) on 7.5% gels and transferred to polyvinylidene fluoride membranes (Millipore, Billercia, MA, USA). The membranes were blocked with TBS containing 5% bovine serum albumin (BSA; Wako). Transferred proteins were detected using a rabbit anti MMP10 polyclonal antibody (ab28205, 1:1000 dilution; Abcam) and mouse anti-β-actin monoclonal antibody (AC-15, 1:5000 dilution; Sigma Aldrich) for an internal control. The membranes were then stained with a peroxidase-labeled secondary antibody and visualized by an enhanced chemiluminescence detection system (Amersham Life Science, Arlington Heights, IL, USA).

In vivo tumorigenicity and limiting dilution assay

Cells (mock, MMP10-overexpressed, si ctrl, si1 and si2) were counted and resuspended at 1 × 10, 1 × 10^2 and 1 × 10^3 cells in 100 µl PBS. 1 × 10 cells were used only for mock and MMP10-overexpressed cells. The suspended cells were mixed with 100 µl Matrigel (BD Biosciences) and injected subcutaneously into the right/left middle back areas of 4 to 6-week-old female nude mice (Charles River Laboratory, Yokohama, Japan) under inhalation anesthesia by isoflurane. Tumor diameters were observed weekly until the mice were sacrificed after 8 to 9 weeks of observation. Tumor volumes were calculated as follows: 0.5 × long axis × short axis of the mass. Stem cell frequencies were calculated in ELDA (Extreme limiting dilution analysis) as described previously (Hu, 2009).

MMP inhibition assay using NNGH

MMP inhibition was performed using NNGH (N-Isobutyl-N-(4-methoxyphenylsufonyl)-glycylhydroxamic acid), an inhibitor of several members of the MMP family including MMP10. NNGH (Santa Cruz Biotechnology, TX, USA) was diluted with DMSO (Dimethyl sulfoxide; Wako) to 0.75 µM and 1.5 µM dilutions, and the same volume of DMSO was used for a control. For the sphere-forming assay, NNGH was added at each dilution to the sphere forming conditioned medium as described above and incubated under shading. Spheres were counted after 7 days of incubation in the same way. For examination of the expression of stem cell-related genes, NNGH was administered at 1.5 µM dilution into spheres that have been cultured for 7 days in sphere forming medium. Cells were collected at 6 hours after administration and the expression of stem cell-related genes was examined.

Patients and specimens

Surgical specimens used for immunohistochemical staining were obtained from 122 patients with primary epithelial ovarian cancer who had been treated at Sapporo Medical University Hospital during the period from 2001 to 2011. A control sample for the endometrium was also obtained from a patient in whom cancer was restricted to the ovary with no invasion to the uterus. Written informed consent was obtained from each patient according to the guidelines of the Declaration of Helsinki. Patients underwent abdominal hysterectomy, bilateral salpingo-oophorectomy, omentum resection, lymphadenectomy and resection of metastatic lesions when possible. All H & E-stained slides were reviewed by pathologist, and the diagnosis was confirmed in accordance with FIGO stage. If adjuvant chemotherapy was needed, almost all cases were administered platinum-based combination agents, and further details of administered agents are shown in table (Table S3). Platinum-resistant cases were defined as cases that progressed with first-line chemotherapy during treatment or relapsed within 6 months after completion of chemotherapy. Optimally resected cases were defined as cases with complete tumor resection or with residual tumor of less than 1 cm in diameter. Overall survival was documented for all patients, and survival was calculated from the day of the operation until November 31, 2013.

Immunohistochemical staining

Sections (5 µm in thickness) of formalin-fixed paraffin-embedded tumors were immunostained using antibodies after epitope retrieval by Novocastra epitope retrieval solution pH 9. To stain specimens, we used the monoclonal antibody of MMP10 (R & D; Clone 110304). The antibody was diluted × 20. Subsequent incubation with a secondary biotynilated antibody was performed, and endogenous peroxidase activity was blocked by immersion in 3% peroxidase. Slides were then counterstained with hematoxylin, rinsed, dehydrated through graded alcohols into a nonaqueous solution, and coverslipped with mounting medium.

Statistical analysis

Statistical analyses were performed with SPSS (version 21 for Windows; SPSS Inc), and GraphPad Prism (version 4.0 for Windows; GraphPad Software Inc) was used for plotting Kaplan-Meier curves. Pearson's χ square tests were used to determine the significance of

associations between characteristic variables. Survival rates were calculated using the Kaplan-Meier method, and differences between groups were tested using the log-rank test. Bilateral Student's t test was performed for examining the statistical difference in qPCR mRNA levels, sphere-forming ability, invading cells in the Matrigel invasion assay, survival rates of chemotherapy-treated cells and *in vivo* tumorigenicity assay. The Cox proportional hazards model was used for multivariate analysis to determine risk ratio and independent significance of individual factors for prognosis. A logistic regression model was used for multivariate analysis to predict odds ratio of individual factors for platinum resistance, and this analysis was performed for cases in advanced stages and cases administered chemotherapies. Each multivariate analysis was performed with the stepwise method. In all analyses, P-values < 0.05 were considered as statistically significant and shown as *< 0.05, **< 0.01.
